# Crystal structure of (*E*)-4-hy­droxy-*N*′-(3-meth­oxy­benzyl­idene)benzohydrazide^1^

**DOI:** 10.1107/S2056989016013268

**Published:** 2016-08-26

**Authors:** Suchada Chantrapromma, Patcharawadee Prachumrat, Pumsak Ruanwas, Nawong Boonnak, Mohammad B. Kassim

**Affiliations:** a Department of Chemistry, Faculty of Science, Prince of Songkla University, Hat-Yai, Songkhla 90112, Thailand; b Department of Basic Science and Mathematics, Faculty of Science, Thaksin University, Muang, Songkhla 90000, Thailand; c School of Chemical Sciences & Food Technology, Faculty of Science & Technology, Universiti Kebangsaan Malaysia, 43600 Bangi, Selangor, Malaysia; d Fuel Cell Institute, Universiti Kebangsaan Malaysia, 43600 Bangi, Selangor, Malaysia

**Keywords:** Benzohydrazides, α-glucosidase inhibitory, X-ray, crystal structure

## Abstract

The title compound, C_15_H_14_N_2_O_3_, crystallizes with two independent mol­ecules (*A* and *B*) in the asymmetric unit that differ in the orientation of the 3-meth­oxy­phenyl group with respect to the methyl­idenebenzohydrazide unit. The dihedral angles between the two benzene rings are 24.02 (10) and 29.30 (9)° in mol­ecules *A* and *B*, respectively. In mol­ecule *A*, the meth­oxy group is twisted slightly relative to its bound benzene ring, with a C_meth­yl_—O—C—C torsion angle of 14.2 (3)°, whereas it is almost co-planar in mol­ecule *B*, where the corresponding angle is −2.4 (3)°. In the crystal, the mol­ecules are linked by N—H⋯O, O—H⋯N and O—H⋯O hydrogen bonds, as well as by weak C—H⋯O inter­actions, forming sheets parallel to the *bc* plane. The N—H⋯O hydrogen bond and weak C—H⋯O inter­action link different mol­ecules (*A*⋯*B*) whereas both O—H⋯N and O—H⋯O hydrogen bonds link like mol­ecules (*A*⋯*A*) and (*B*⋯*B*). Pairs of inversion-related *B* mol­ecules are stacked approximately along the *a* axis by π–π inter­actions in which the distance between the centroids of the 3-meth­oxy­phenyl rings is 3.5388 (12) Å. The *B* mol­ecules also participate in weak C—H⋯π inter­actions between the 4-hy­droxy­phenyl and the 3-meth­oxy­phenyl rings.

## Abstract 2

The mol­ecules of C_15_H_14_N_2_O_3_ are slightly twisted. N—H⋯O, O—H⋯N and O—H⋯O hydrogen bonds play an important role in the crystal packing, resulting in the formation of mol­ecular sheets parallel to the *bc* plane.

## Chemical context   

The benzohydrazide pharmacophore, which comprises >C=O, >C=N– and >NH groups, has attracted much attention from medicinal chemists as a result of its important biological properties. Various derivatives of benzohydrazide have been reported to possess a range of biological properties, including anti­bacterial (Bhole & Bhusari, 2009 ▸; Peng, 2011 ▸), anti­fungal (Loncle *et al.*, 2004 ▸), anti­tubercular (Bedia *et al.*, 2006 ▸) and anti­malarial activities (Melnyk *et al.*, 2006 ▸). Recently, α-glucosidase inhibitory activity of benzohydrazides has been reported (Imran *et al.*, 2015 ▸; Taha *et al.*, 2015 ▸).

**Figure 1:**
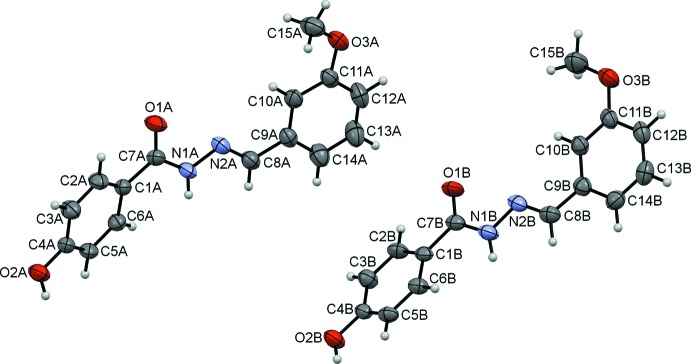
The mol­ecular structure of (I), showing 50% probability displacement ellipsoids and the atom-numbering scheme.

The inter­esting biological activities of benzohydrazides led us to synthesize the title compound (I) and study its α-glucosidase inhibitory activity. The result indicates that (I) possesses weak α-glucosidase inhibitory activity with 7.30±2.85% inhibition at a concentration of 100 µg/mL. The structure of (I) was characterized by spectroscopy while its X-ray structure, Fig. 1 ▸, confirms the formation of the *N*′-benzyl­idenebenzohydrazide skeleton. In our previous studies, we reported the syntheses and crystal structures of two related compounds, (*E*)-4-hy­droxy-*N*′-(3-hy­droxy-4-meth­oxy­benzyl­idene)benzohydrazide (Fun *et al.*, 2011 ▸) and (*E*)-4-hy­droxy-*N*′-(3,4,5-tri­meth­oxy­benzyl­idene)benzohydrazide (Horkaew *et al.*, 2011 ▸).
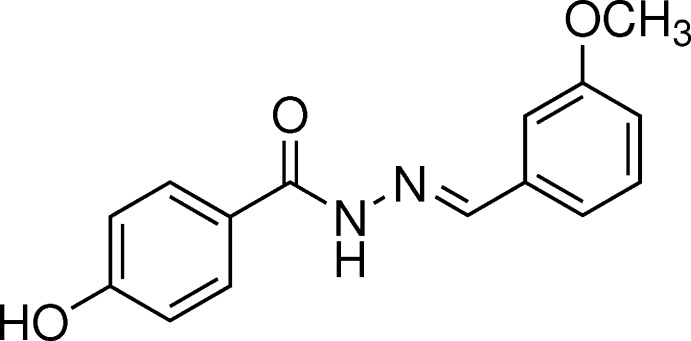


## Structural commentary   

There are two crystallographically independent mol­ecules, *A* and *B*, of the title benzohydrazide derivative, C_15_H_14_N_2_O_3_, in the asymmetric unit of (I). These differ in the orientation of the 3-meth­oxy­phenyl ring with respect to the methyl­idene­benzo­hydrazide unit. The dihedral angles between the two benzene rings are 24.02 (10) and 29.30 (9)° in mol­ecules *A* and *B*, respectively. The mol­ecules exist in the *trans*-conformation with respect to the C8=N2 bond [1.275 (2) Å in mol­ecule *A* and 1.271 (2) Å in mol­ecule *B*] and the torsion angle N1—N2—C8—C9 = −178.14 (16)° in mol­ecule *A* and −177.69 (16)° in mol­ecule *B*. Five atoms (O1, C7, N1, N2 and C8) of the central fragment are approximately coplanar, having r.m.s. deviations of 0.0179 (2) Å in mol­ecule *A* and 0.0327 (2) Å in mol­ecule *B*. The mean plane through this central fragment makes dihedral angles of 23.87 (11) and 0.20 (12)° with the planes of the 4-hy­droxy­phenyl and 3-meth­oxy­phenyl rings, respectively, in mol­ecule *A*. The corresponding values are 22.58 (11) and 11.04 (11) ° in mol­ecule *B*. In mol­ecule *A*, the meth­oxy group is slightly twisted from the attached benzene ring [C15—O3—C11—C10 = 14.2 (3)°] whereas it is essentially coplanar in mol­ecule *B* [where the corresponding torsion angle is −2.4 (3)°]. The bond distances agree with literature values and are comparable with those in related structures (Fun *et al.*, 2011 ▸; Horkaew *et al.*, 2011 ▸; Rassem *et al.*, 2012 ▸; Shi, 2009 ▸).

## Supra­molecular features   

**Figure 2:**
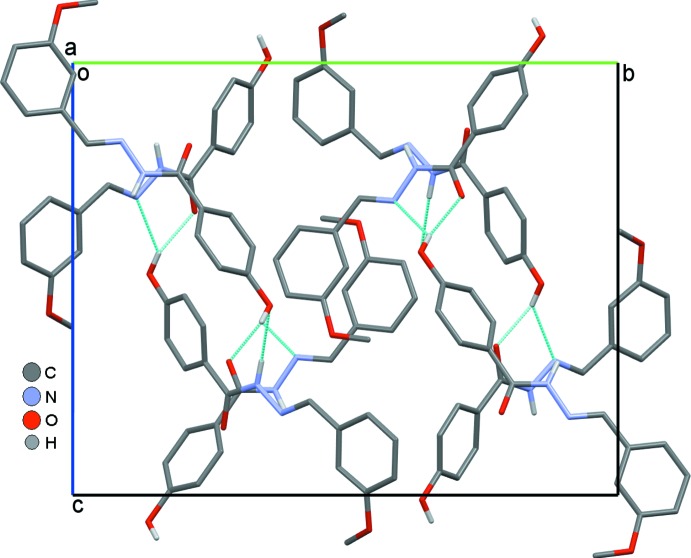
Mol­ecular packing of (I) linked by N—H⋯O, O—H⋯N and O—H⋯O hydrogen bonds drawn as dotted lines.

**Figure 3:**
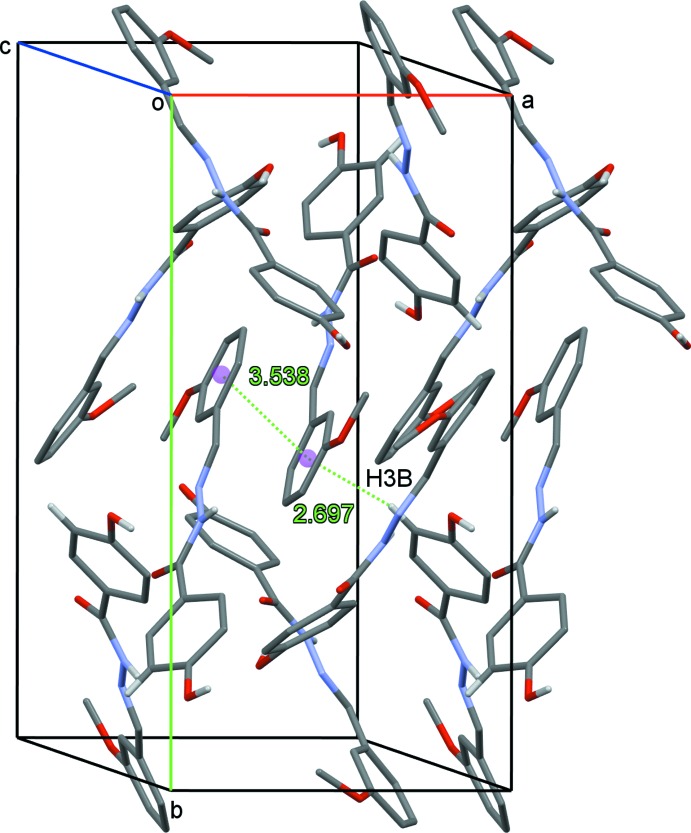
C—H⋯π and π–π contacts for (I) drawn as dotted lines with the centroids of the C9*B*–C14*B* rings centroids shown as coloured spheres.

In the crystal (Fig. 2 ▸), the mol­ecules are linked by N—H⋯O, O—H⋯N and O—H⋯O hydrogen bonds, as well as by weak C—H⋯O inter­actions (Table 1 ▸), into sheets parallel to the *bc* plane. The N1*A*—H1*A*⋯O2*B* ^i^ and N1*B*—H1*B*⋯O3*A* ^ii^ hydrogen bonds and C13*B*—H13*B*⋯O1*A* ^iv^ inter­actions link non-equivalent mol­ecules (*A*⋯*B*) whereas the O2*A*—H2*A*⋯N2*A* ^iii^ and O2*A*—H2*A*⋯O1*A* ^iii^ hydrogen bonds link equivalent *A* mol­ecules, and O2*B*—H2*B*⋯N2*B* ^ii^ and O2*B*—H2*B*⋯O1*B* ^ii^ hydrogen bonds link equivalent *B* mol­ecules. Stacking of planes of mol­ecules in the *a*-axis direction involves π–π inter­actions between *B* mol­ecules with *Cg*⋯*Cg* ^vi^ distance of 3.5388 (12) Å. A weak C—H⋯π inter­action (C3*B*—H3*B*⋯*Cg* ^v^) between the 4-hy­droxy­phenyl ring and the 3-meth­oxy­phenyl ring of symmetry-related *B* mol­ecules is also present (Fig. 3 ▸, Table 1 ▸) [symmetry codes: (i) −*x*, 1 − *y*, 1 − *z*; (ii) *x*, 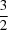 − *y*, −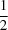 + *z*; (iii) *x*, 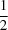 − *y*, −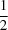 + *z*; (iv) *x*, 1 + *y*, *z*; (v) −*x*, −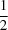 + *y*, 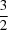 − *z*; (vi) 1 − *x*, 2 − *y*, 2 − *z*; *Cg* is the centroid of the C9*B*–C14*B* ring].

## Database survey   

A search of SciFinder (Scifinder, 2015 ▸) reveals a total of 719 related structures with benzohydrazides, and 52 related structure with 4-hy­droxy­benzohydrazides. Specific examples by Fun *et al.*, 2011 ▸; Horkaew *et al.*, 2011 ▸; Rassem *et al.*, 2012 ▸; Shi, 2009 ▸) have been mentioned in the *Chemical context* section.

## Synthesis and crystallization   

A solution of 4-hy­droxy­benzohydrazide (2 mmol, 0.30 g) in ethanol (10 ml) and 3-meth­oxy­benzaldehyde (2 mmol, 0.27 g) in ethanol (10 ml) were mixed, stirred and refluxed for 5 h. The resulting mixture was then cooled to room temperature. The white precipitate that formed was filtered. Colorless block-shaped single crystals of (I) suitable for X-ray structure determination were recrystallized from methanol by slow evaporation at room temperature over a period of several days, m.p. 478–479 K.

## Spectroscopic studies and α-glucosidase inhibitory assay   

UV–Vis (CH_3_OH) λ_max_ (log∊): 212 (5.51), 302 (5.61) nm; FT–IR (KBr) ν: 3158, 2834, 1648, 1607, 1509 cm^−1^; ^1^H NMR (300 MHz, DMSO-*d*_6_) δ: 11.65 (*s*, 1H, NH), 10.15 (*s*, 1H, Ar—OH), 8.39 (*s*, 1H, N=CH), 7.80 (*d*, *J* = 8.7 Hz, 2H, Ar—H), 7.27 (*s*, 1H, Ar—H), 7.25 (*br d*, *J* = 8.4 Hz, 1H, Ar—H), 7.37 (*t*, *J* = 8.4 Hz, 1H, Ar—H), 7.00 (*br d*, *J* = 8.4 Hz, 1H, Ar—H), 6.86 (*d*, *J* = 8.7 Hz, 2H, Ar—H), 3.81 (*s*, 3H, –OCH3) p.p.m.

The UV–Vis spectrum of (I) shows absorption bands of a benzohydrazide (212 and 302 nm). The IR spectrum of (I) shows the typical stretching of C=N and amide C=O functionalities at 1648 and 1607 cm^−1^, respectively, which confirm the successful synthesis of the *N*′-benzyl­idenebenzohydrazide skeleton. In addition, the ^1^H NMR spectrum of (I) also supports the formation of the *N*′-benzyl­idenebenzohydrazide skeleton by showing the characteristic signals of an amine (N=CH) at 8.39 (*s*, 1H) and an amide (N—H) at 11.65 (*s*, 1H) p.p.m.

The α-glucosidase inhibitory assay was modified from the method of Kim *et al.* (2004 ▸). The result showed that (I) possesses weak activity with 7.30±2.85% inhibition at a concentration of 100 µg/mL.

## Refinement   

Crystal data, data collection and structure refinement details are summarized in Table 2 ▸. Hydrogen atoms were positioned geometrically and allowed to ride on their parent atoms, with *d*(N—H) = 0.85 or 0.87 Å; d(O—H) = 0.82 Å; d(C—H) = 0.93 Å for aromatic and CH; and 0.96 Å for CH_3_ atoms. The *U*_iso_ values were constrained to be 1.5*U*_eq_ of the carrier atom for methyl and hydroxyl H atoms, and 1.2*U*_eq_ for the remaining H atoms. A rotating group model was used for the methyl groups.

## Supplementary Material

Crystal structure: contains datablock(s) global, I. DOI: 10.1107/S2056989016013268/pk2585sup1.cif

Structure factors: contains datablock(s) I. DOI: 10.1107/S2056989016013268/pk2585Isup2.hkl

Supporting information file. DOI: 10.1107/S2056989016013268/pk2585Isup3.cml

CCDC reference: 1499671

Additional supporting information: crystallographic information; 3D view; checkCIF report

## Figures and Tables

**Table 1:** Hydrogen-bond geometry (Å, °) *Cg*4 is the centroid of the C9*B*–C14*B* ring.

*D*—H⋯*A*	*D*—H	H⋯*A*	*D*⋯*A*	*D*—H⋯*A*
N1*A*—H1*A*⋯O2*B* ^i^	0.85	2.58	3.354 (2)	153
N1*B*—H1*B*⋯O3*A* ^ii^	0.87	2.32	3.178 (3)	170
O2*A*—H2*A*⋯O1*A* ^iii^	0.82	1.94	2.702 (2)	155
O2*A*—H2*A*⋯N2*A* ^iii^	0.82	2.60	3.231 (2)	135
O2*B*—H2*B*⋯O1*B* ^ii^	0.82	1.92	2.696 (2)	157
O2*B*—H2*B*⋯N2*B* ^ii^	0.82	2.52	3.110 (2)	129
C13*B*—H13*B*⋯O1*A* ^iv^	0.93	2.57	3.352 (3)	143
C3*B*—H3*B*⋯*Cg* ^v^	0.93	2.70	3.604 (2)	165

Symmetry codes: (i) 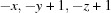; (ii) 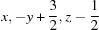; (iii) 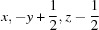; (iv) 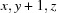; (v) 
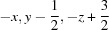
.

**Table 2:** Experimental details

Crystal data	
Chemical formula	C_15_H_14_N_2_O_3_
*M*_r_	270.28
Crystal system, space group	Monoclinic, *P*2_1_/*c*
Temperature (K)	300
*a*, *b*, *c* (Å)	9.2713 (6), 19.0235 (11), 15.6054 (9)
β (°)	105.118 (2)
*V* (Å^3^)	2657.1 (3)
*Z*	8
Radiation type	Mo *K*α
μ (mm^−1^)	0.10
Crystal size (mm)	0.13 × 0.10 × 0.10

Data collection	
Diffractometer	Bruker *SMART*
Absorption correction	Multi-scan (*SADABS*; Bruker, 2007 ▸)
*T*_min_, *T*_max_	0.988, 0.991
No. of measured, independent and observed [*I* > 2σ(*I*)] reflections	70844, 5213, 3311
*R*_int_	0.103
(sin θ/λ)_max_ (Å^−1^)	0.617

Refinement	
*R*[*F* ^2^ > 2σ(*F* ^2^)], *wR*(*F* ^2^), *S*	0.046, 0.105, 1.06
No. of reflections	5213
No. of parameters	364
H-atom treatment	H-atom parameters constrained
Δρ_max_, Δρ_min_ (e Å^−3^)	0.14, −0.16

Computer programs: *SMART* and *SAINT* (Bruker, 2007 ▸), *Mercury* (Macrae *et al.*, 2006 ▸), *SHELXTL* (Sheldrick, 2008 ▸), *PLATON* (Spek, 2009 ▸) and *publCIF* (Westrip, 2010 ▸).

## supplementary crystallographic information

### Crystal data

**Table:**

C_15_H_14_N_2_O_3_	*D*_x_ = 1.351 Mg m^−^^3^
*M**_r_* = 270.28	Melting point = 478–479 K
Monoclinic, *P*2_1_/*c*	Mo *K*α radiation, λ = 0.71073 Å
*a* = 9.2713 (6) Å	Cell parameters from 5213 reflections
*b* = 19.0235 (11) Å	θ = 2.9–26.0°
*c* = 15.6054 (9) Å	µ = 0.10 mm^−^^1^
β = 105.118 (2)°	*T* = 300 K
*V* = 2657.1 (3) Å^3^	Block, colorless
*Z* = 8	0.13 × 0.10 × 0.10 mm
*F*(000) = 1136	

### Data collection

**Table:**

Bruker SMART diffractometer	3311 reflections with *I* > 2σ(*I*)
φ and ω scans	*R*_int_ = 0.103
Absorption correction: multi-scan (SADABS; Bruker, 2007)	θ_max_ = 26.0°, θ_min_ = 2.9°
*T*_min_ = 0.988, *T*_max_ = 0.991	*h* = −11→11
70844 measured reflections	*k* = −23→23
5213 independent reflections	*l* = −19→18

### Refinement

**Table:**

Refinement on *F*^2^	Secondary atom site location: difference Fourier map
Least-squares matrix: full	Hydrogen site location: inferred from neighbouring sites
*R*[*F*^2^ > 2σ(*F*^2^)] = 0.046	H-atom parameters constrained
*wR*(*F*^2^) = 0.105	*w* = 1/[σ^2^(*F*_o_^2^) + (0.040*P*)^2^ + 0.602*P*] where *P* = (*F*_o_^2^ + 2*F*_c_^2^)/3
*S* = 1.06	(Δ/σ)_max_ = 0.001
5213 reflections	Δρ_max_ = 0.14 e Å^−^^3^
364 parameters	Δρ_min_ = −0.16 e Å^−^^3^
0 restraints	Extinction correction: SHELXL, Fc^*^=kFc[1+0.001xFc^2^λ^3^/sin(2θ)]^-1/4^
Primary atom site location: structure-invariant direct methods	Extinction coefficient: 0.0036 (6)

### Special details

**Table:**

Geometry. All esds (except the esd in the dihedral angle between two l.s. planes) are estimated using the full covariance matrix. The cell esds are taken into account individually in the estimation of esds in distances, angles and torsion angles; correlations between esds in cell parameters are only used when they are defined by crystal symmetry. An approximate (isotropic) treatment of cell esds is used for estimating esds involving l.s. planes.
Refinement. Refinement of F^2^ against ALL reflections. The weighted R-factor wR and goodness of fit S are based on F^2^, conventional R-factors R are based on F, with F set to zero for negative F^2^. The threshold expression of F^2^ > 2sigma(F^2^) is used only for calculating R-factors(gt) etc. and is not relevant to the choice of reflections for refinement. R-factors based on F^2^ are statistically about twice as large as those based on F, and R- factors based on ALL data will be even larger.

### Fractional atomic coordinates and isotropic or equivalent isotropic displacement parameters (Å^2^)

**Table:**

	*x*	*y*	*z*	*U*_iso_*/*U*_eq_	
O1A	0.43409 (17)	0.27645 (8)	0.84360 (9)	0.0583 (4)	
O2A	0.50983 (17)	0.14048 (8)	0.48887 (9)	0.0572 (4)	
H2A	0.4658	0.1573	0.4409	0.086*	
O3A	0.25586 (17)	0.54386 (7)	1.08045 (9)	0.0536 (4)	
N1A	0.26003 (19)	0.33795 (8)	0.74482 (10)	0.0447 (4)	
H1A	0.2151	0.3461	0.6911	0.054*	
N2A	0.24503 (19)	0.38366 (9)	0.81022 (10)	0.0445 (4)	
C1A	0.3853 (2)	0.24216 (9)	0.69253 (12)	0.0352 (4)	
C2A	0.5205 (2)	0.20673 (10)	0.70688 (13)	0.0436 (5)	
H2A1	0.5851	0.2058	0.7636	0.052*	
C3A	0.5603 (2)	0.17300 (11)	0.63865 (13)	0.0471 (5)	
H3A	0.6518	0.1501	0.6492	0.057*	
C4A	0.4647 (2)	0.17309 (9)	0.55460 (12)	0.0386 (5)	
C5A	0.3262 (2)	0.20502 (10)	0.53997 (12)	0.0416 (5)	
H5A	0.2594	0.2033	0.4840	0.050*	
C6A	0.2875 (2)	0.23933 (10)	0.60853 (12)	0.0398 (5)	
H6A	0.1945	0.2609	0.5983	0.048*	
C7A	0.3611 (2)	0.28530 (10)	0.76666 (12)	0.0392 (5)	
C8A	0.1550 (2)	0.43474 (10)	0.78455 (13)	0.0440 (5)	
H8AA	0.1032	0.4378	0.7250	0.053*	
C9A	0.1313 (2)	0.48859 (10)	0.84627 (13)	0.0421 (5)	
C10A	0.2082 (2)	0.48732 (10)	0.93602 (13)	0.0432 (5)	
H10A	0.2769	0.4518	0.9579	0.052*	
C11A	0.1817 (2)	0.53895 (10)	0.99222 (13)	0.0439 (5)	
C12A	0.0746 (3)	0.58989 (11)	0.96013 (16)	0.0560 (6)	
H12A	0.0518	0.6228	0.9987	0.067*	
C13A	0.0025 (3)	0.59198 (12)	0.87190 (17)	0.0625 (6)	
H13A	−0.0670	0.6272	0.8505	0.075*	
C14A	0.0317 (2)	0.54234 (11)	0.81427 (15)	0.0550 (6)	
H14A	−0.0155	0.5450	0.7540	0.066*	
C15A	0.3876 (3)	0.50331 (13)	1.10988 (15)	0.0653 (7)	
H15A	0.3614	0.4549	1.1149	0.098*	
H15B	0.4440	0.5202	1.1667	0.098*	
H15C	0.4467	0.5073	1.0680	0.098*	
O1B	0.09195 (17)	0.78644 (7)	0.80813 (9)	0.0548 (4)	
O2B	0.02120 (15)	0.63829 (7)	0.43779 (9)	0.0514 (4)	
H2B	0.0628	0.6534	0.4012	0.077*	
O3B	0.33565 (17)	1.02397 (7)	1.12977 (9)	0.0571 (4)	
N1B	0.19818 (18)	0.87025 (9)	0.74304 (10)	0.0455 (4)	
H1B	0.2149	0.8885	0.6952	0.055*	
N2B	0.22473 (18)	0.91065 (9)	0.81927 (10)	0.0427 (4)	
C1B	0.1108 (2)	0.76347 (10)	0.66236 (11)	0.0356 (4)	
C2B	0.0026 (2)	0.71118 (10)	0.64794 (12)	0.0419 (5)	
H2B1	−0.0523	0.7042	0.6893	0.050*	
C3B	−0.0245 (2)	0.66956 (11)	0.57357 (13)	0.0443 (5)	
H3B	−0.0972	0.6347	0.5650	0.053*	
C4B	0.0561 (2)	0.67953 (10)	0.51149 (12)	0.0364 (4)	
C5B	0.1669 (2)	0.72987 (10)	0.52587 (12)	0.0395 (5)	
H5B	0.2234	0.7357	0.4851	0.047*	
C6B	0.1938 (2)	0.77149 (10)	0.60057 (12)	0.0391 (5)	
H6B	0.2685	0.8054	0.6098	0.047*	
C7B	0.1325 (2)	0.80650 (10)	0.74351 (12)	0.0398 (5)	
C8B	0.2720 (2)	0.97284 (11)	0.81420 (13)	0.0444 (5)	
H8BA	0.2831	0.9889	0.7600	0.053*	
C9B	0.3094 (2)	1.01972 (10)	0.89046 (13)	0.0406 (5)	
C10B	0.2934 (2)	0.99840 (10)	0.97275 (13)	0.0413 (5)	
H10B	0.2493	0.9553	0.9784	0.050*	
C11B	0.3427 (2)	1.04106 (10)	1.04576 (13)	0.0432 (5)	
C12B	0.4064 (2)	1.10571 (10)	1.03710 (15)	0.0487 (5)	
H12B	0.4415	1.1342	1.0866	0.058*	
C13B	0.4175 (2)	1.12758 (11)	0.95555 (16)	0.0524 (6)	
H13B	0.4581	1.1715	0.9496	0.063*	
C14B	0.3690 (2)	1.08499 (11)	0.88184 (14)	0.0486 (5)	
H14B	0.3764	1.1003	0.8266	0.058*	
C15B	0.2675 (3)	0.95877 (13)	1.14034 (16)	0.0703 (7)	
H15D	0.1643	0.9597	1.1077	0.105*	
H15E	0.2746	0.9511	1.2021	0.105*	
H15F	0.3177	0.9214	1.1184	0.105*	

### Atomic displacement parameters (Å^2^)

**Table:**

	*U*^11^	*U*^22^	*U*^33^	*U*^12^	*U*^13^	*U*^23^
O1A	0.0767 (11)	0.0669 (10)	0.0272 (8)	0.0185 (8)	0.0063 (7)	−0.0019 (7)
O2A	0.0749 (10)	0.0647 (10)	0.0332 (8)	0.0202 (8)	0.0163 (7)	−0.0020 (7)
O3A	0.0674 (10)	0.0563 (9)	0.0421 (9)	0.0040 (8)	0.0233 (8)	−0.0093 (7)
N1A	0.0599 (11)	0.0465 (10)	0.0263 (8)	0.0091 (9)	0.0087 (8)	−0.0062 (7)
N2A	0.0578 (11)	0.0449 (10)	0.0343 (9)	0.0003 (9)	0.0180 (8)	−0.0083 (8)
C1A	0.0442 (11)	0.0326 (10)	0.0285 (10)	−0.0021 (9)	0.0088 (8)	0.0007 (8)
C2A	0.0470 (12)	0.0498 (12)	0.0290 (11)	0.0036 (10)	0.0010 (9)	−0.0024 (9)
C3A	0.0455 (12)	0.0531 (13)	0.0401 (12)	0.0110 (10)	0.0064 (10)	−0.0024 (10)
C4A	0.0535 (13)	0.0344 (11)	0.0291 (10)	0.0023 (9)	0.0130 (9)	0.0003 (8)
C5A	0.0523 (13)	0.0412 (11)	0.0269 (10)	0.0025 (10)	0.0026 (9)	−0.0007 (9)
C6A	0.0418 (11)	0.0401 (11)	0.0350 (11)	0.0064 (9)	0.0055 (9)	0.0015 (9)
C7A	0.0483 (12)	0.0409 (11)	0.0298 (11)	−0.0014 (10)	0.0125 (9)	0.0010 (9)
C8A	0.0493 (12)	0.0455 (12)	0.0389 (11)	−0.0026 (10)	0.0144 (10)	−0.0063 (10)
C9A	0.0450 (12)	0.0403 (11)	0.0441 (12)	−0.0050 (10)	0.0169 (10)	−0.0082 (9)
C10A	0.0502 (12)	0.0407 (11)	0.0442 (12)	0.0009 (9)	0.0222 (10)	−0.0020 (9)
C11A	0.0515 (13)	0.0434 (12)	0.0419 (12)	−0.0077 (10)	0.0212 (10)	−0.0078 (10)
C12A	0.0589 (14)	0.0465 (13)	0.0655 (16)	0.0012 (11)	0.0215 (12)	−0.0200 (11)
C13A	0.0578 (15)	0.0512 (14)	0.0730 (17)	0.0118 (11)	0.0072 (13)	−0.0147 (13)
C14A	0.0520 (14)	0.0534 (14)	0.0555 (14)	0.0007 (11)	0.0071 (11)	−0.0116 (11)
C15A	0.0728 (16)	0.0820 (17)	0.0445 (14)	0.0122 (14)	0.0212 (12)	−0.0030 (12)
O1B	0.0828 (11)	0.0569 (9)	0.0305 (8)	−0.0083 (8)	0.0249 (8)	−0.0005 (7)
O2B	0.0628 (9)	0.0577 (9)	0.0376 (8)	−0.0053 (7)	0.0203 (7)	−0.0119 (7)
O3B	0.0770 (11)	0.0564 (10)	0.0427 (9)	−0.0050 (8)	0.0243 (8)	−0.0114 (7)
N1B	0.0596 (11)	0.0526 (11)	0.0263 (9)	−0.0061 (9)	0.0149 (8)	−0.0037 (8)
N2B	0.0491 (10)	0.0508 (11)	0.0275 (9)	0.0003 (8)	0.0090 (7)	−0.0048 (8)
C1B	0.0400 (11)	0.0418 (11)	0.0240 (10)	0.0045 (9)	0.0067 (8)	0.0021 (8)
C2B	0.0465 (12)	0.0522 (12)	0.0321 (11)	−0.0004 (10)	0.0194 (9)	0.0011 (9)
C3B	0.0473 (12)	0.0485 (12)	0.0400 (12)	−0.0057 (10)	0.0165 (10)	−0.0053 (10)
C4B	0.0421 (11)	0.0407 (11)	0.0263 (10)	0.0067 (9)	0.0089 (9)	−0.0011 (8)
C5B	0.0414 (11)	0.0504 (12)	0.0304 (10)	0.0037 (10)	0.0161 (9)	0.0037 (9)
C6B	0.0408 (11)	0.0469 (12)	0.0299 (10)	−0.0016 (9)	0.0099 (9)	0.0014 (9)
C7B	0.0440 (12)	0.0480 (12)	0.0269 (11)	0.0021 (10)	0.0081 (9)	0.0022 (9)
C8B	0.0486 (12)	0.0520 (13)	0.0348 (11)	0.0006 (10)	0.0148 (10)	0.0014 (9)
C9B	0.0396 (11)	0.0419 (12)	0.0405 (12)	0.0040 (9)	0.0106 (9)	−0.0017 (9)
C10B	0.0433 (11)	0.0399 (11)	0.0431 (12)	−0.0001 (9)	0.0159 (9)	−0.0046 (9)
C11B	0.0457 (12)	0.0409 (12)	0.0445 (12)	0.0064 (10)	0.0144 (10)	−0.0078 (10)
C12B	0.0503 (13)	0.0395 (12)	0.0540 (14)	0.0053 (10)	0.0098 (11)	−0.0118 (10)
C13B	0.0524 (13)	0.0350 (12)	0.0694 (16)	0.0015 (10)	0.0150 (12)	0.0001 (11)
C14B	0.0548 (13)	0.0449 (13)	0.0485 (13)	0.0077 (10)	0.0177 (11)	0.0087 (10)
C15B	0.099 (2)	0.0647 (16)	0.0586 (16)	−0.0104 (15)	0.0407 (14)	−0.0043 (13)

### Geometric parameters (Å, º)

**Table:**

O1A—C7A	1.226 (2)	O1B—C7B	1.225 (2)
O2A—C4A	1.355 (2)	O2B—C4B	1.360 (2)
O2A—H2A	0.8194	O2B—H2B	0.8198
O3A—C11A	1.372 (2)	O3B—C11B	1.369 (2)
O3A—C15A	1.416 (3)	O3B—C15B	1.421 (3)
N1A—C7A	1.353 (2)	N1B—C7B	1.358 (2)
N1A—N2A	1.375 (2)	N1B—N2B	1.383 (2)
N1A—H1A	0.8478	N1B—H1B	0.8736
N2A—C8A	1.275 (2)	N2B—C8B	1.271 (2)
C1A—C6A	1.387 (2)	C1B—C2B	1.388 (3)
C1A—C2A	1.389 (3)	C1B—C6B	1.390 (2)
C1A—C7A	1.483 (3)	C1B—C7B	1.477 (3)
C2A—C3A	1.373 (3)	C2B—C3B	1.373 (3)
C2A—H2A1	0.9300	C2B—H2B1	0.9300
C3A—C4A	1.378 (3)	C3B—C4B	1.382 (3)
C3A—H3A	0.9300	C3B—H3B	0.9300
C4A—C5A	1.385 (3)	C4B—C5B	1.379 (3)
C5A—C6A	1.378 (3)	C5B—C6B	1.377 (2)
C5A—H5A	0.9300	C5B—H5B	0.9300
C6A—H6A	0.9300	C6B—H6B	0.9300
C8A—C9A	1.461 (3)	C8B—C9B	1.455 (3)
C8A—H8AA	0.9300	C8B—H8BA	0.9300
C9A—C14A	1.381 (3)	C9B—C14B	1.380 (3)
C9A—C10A	1.396 (3)	C9B—C10B	1.391 (3)
C10A—C11A	1.381 (3)	C10B—C11B	1.376 (3)
C10A—H10A	0.9300	C10B—H10B	0.9300
C11A—C12A	1.384 (3)	C11B—C12B	1.386 (3)
C12A—C13A	1.365 (3)	C12B—C13B	1.368 (3)
C12A—H12A	0.9300	C12B—H12B	0.9300
C13A—C14A	1.379 (3)	C13B—C14B	1.383 (3)
C13A—H13A	0.9300	C13B—H13B	0.9300
C14A—H14A	0.9300	C14B—H14B	0.9300
C15A—H15A	0.9600	C15B—H15D	0.9600
C15A—H15B	0.9600	C15B—H15E	0.9600
C15A—H15C	0.9600	C15B—H15F	0.9600
			
C4A—O2A—H2A	109.6	C4B—O2B—H2B	109.5
C11A—O3A—C15A	116.91 (15)	C11B—O3B—C15B	116.88 (16)
C7A—N1A—N2A	118.59 (16)	C7B—N1B—N2B	118.10 (16)
C7A—N1A—H1A	120.9	C7B—N1B—H1B	122.7
N2A—N1A—H1A	120.0	N2B—N1B—H1B	118.9
C8A—N2A—N1A	115.67 (16)	C8B—N2B—N1B	116.77 (16)
C6A—C1A—C2A	118.28 (17)	C2B—C1B—C6B	118.26 (17)
C6A—C1A—C7A	124.35 (17)	C2B—C1B—C7B	117.84 (16)
C2A—C1A—C7A	117.18 (16)	C6B—C1B—C7B	123.90 (18)
C3A—C2A—C1A	121.01 (18)	C3B—C2B—C1B	121.04 (17)
C3A—C2A—H2A1	119.5	C3B—C2B—H2B1	119.5
C1A—C2A—H2A1	119.5	C1B—C2B—H2B1	119.5
C2A—C3A—C4A	120.08 (19)	C2B—C3B—C4B	119.98 (19)
C2A—C3A—H3A	120.0	C2B—C3B—H3B	120.0
C4A—C3A—H3A	120.0	C4B—C3B—H3B	120.0
O2A—C4A—C3A	118.10 (18)	O2B—C4B—C5B	122.75 (16)
O2A—C4A—C5A	122.16 (17)	O2B—C4B—C3B	117.43 (17)
C3A—C4A—C5A	119.73 (17)	C5B—C4B—C3B	119.81 (17)
C6A—C5A—C4A	119.84 (17)	C6B—C5B—C4B	120.00 (17)
C6A—C5A—H5A	120.1	C6B—C5B—H5B	120.0
C4A—C5A—H5A	120.1	C4B—C5B—H5B	120.0
C5A—C6A—C1A	120.91 (18)	C5B—C6B—C1B	120.86 (18)
C5A—C6A—H6A	119.5	C5B—C6B—H6B	119.6
C1A—C6A—H6A	119.5	C1B—C6B—H6B	119.6
O1A—C7A—N1A	121.15 (17)	O1B—C7B—N1B	121.12 (17)
O1A—C7A—C1A	122.06 (18)	O1B—C7B—C1B	122.05 (18)
N1A—C7A—C1A	116.69 (16)	N1B—C7B—C1B	116.82 (16)
N2A—C8A—C9A	121.71 (19)	N2B—C8B—C9B	122.14 (18)
N2A—C8A—H8AA	119.1	N2B—C8B—H8BA	118.9
C9A—C8A—H8AA	119.1	C9B—C8B—H8BA	118.9
C14A—C9A—C10A	119.58 (18)	C14B—C9B—C10B	119.63 (18)
C14A—C9A—C8A	118.82 (19)	C14B—C9B—C8B	119.20 (19)
C10A—C9A—C8A	121.59 (19)	C10B—C9B—C8B	121.09 (18)
C11A—C10A—C9A	119.74 (19)	C11B—C10B—C9B	119.99 (19)
C11A—C10A—H10A	120.1	C11B—C10B—H10B	120.0
C9A—C10A—H10A	120.1	C9B—C10B—H10B	120.0
O3A—C11A—C10A	124.05 (19)	O3B—C11B—C10B	124.36 (18)
O3A—C11A—C12A	116.09 (18)	O3B—C11B—C12B	115.60 (18)
C10A—C11A—C12A	119.9 (2)	C10B—C11B—C12B	120.03 (19)
C13A—C12A—C11A	120.1 (2)	C13B—C12B—C11B	119.9 (2)
C13A—C12A—H12A	119.9	C13B—C12B—H12B	120.1
C11A—C12A—H12A	119.9	C11B—C12B—H12B	120.1
C12A—C13A—C14A	120.7 (2)	C12B—C13B—C14B	120.5 (2)
C12A—C13A—H13A	119.7	C12B—C13B—H13B	119.7
C14A—C13A—H13A	119.7	C14B—C13B—H13B	119.7
C13A—C14A—C9A	119.9 (2)	C9B—C14B—C13B	119.9 (2)
C13A—C14A—H14A	120.1	C9B—C14B—H14B	120.1
C9A—C14A—H14A	120.1	C13B—C14B—H14B	120.1
O3A—C15A—H15A	109.5	O3B—C15B—H15D	109.5
O3A—C15A—H15B	109.5	O3B—C15B—H15E	109.5
H15A—C15A—H15B	109.5	H15D—C15B—H15E	109.5
O3A—C15A—H15C	109.5	O3B—C15B—H15F	109.5
H15A—C15A—H15C	109.5	H15D—C15B—H15F	109.5
H15B—C15A—H15C	109.5	H15E—C15B—H15F	109.5
			
C7A—N1A—N2A—C8A	175.70 (18)	C7B—N1B—N2B—C8B	−172.99 (18)
C6A—C1A—C2A—C3A	3.6 (3)	C6B—C1B—C2B—C3B	−1.6 (3)
C7A—C1A—C2A—C3A	−171.59 (18)	C7B—C1B—C2B—C3B	179.43 (18)
C1A—C2A—C3A—C4A	−0.9 (3)	C1B—C2B—C3B—C4B	−0.2 (3)
C2A—C3A—C4A—O2A	178.58 (18)	C2B—C3B—C4B—O2B	−177.96 (17)
C2A—C3A—C4A—C5A	−2.4 (3)	C2B—C3B—C4B—C5B	2.0 (3)
O2A—C4A—C5A—C6A	−178.03 (18)	O2B—C4B—C5B—C6B	177.99 (17)
C3A—C4A—C5A—C6A	3.0 (3)	C3B—C4B—C5B—C6B	−2.0 (3)
C4A—C5A—C6A—C1A	−0.3 (3)	C4B—C5B—C6B—C1B	0.1 (3)
C2A—C1A—C6A—C5A	−3.0 (3)	C2B—C1B—C6B—C5B	1.6 (3)
C7A—C1A—C6A—C5A	171.83 (17)	C7B—C1B—C6B—C5B	−179.48 (17)
N2A—N1A—C7A—O1A	2.8 (3)	N2B—N1B—C7B—O1B	3.0 (3)
N2A—N1A—C7A—C1A	−173.49 (16)	N2B—N1B—C7B—C1B	−177.75 (16)
C6A—C1A—C7A—O1A	165.80 (19)	C2B—C1B—C7B—O1B	20.7 (3)
C2A—C1A—C7A—O1A	−19.4 (3)	C6B—C1B—C7B—O1B	−158.18 (19)
C6A—C1A—C7A—N1A	−17.9 (3)	C2B—C1B—C7B—N1B	−158.50 (17)
C2A—C1A—C7A—N1A	156.92 (17)	C6B—C1B—C7B—N1B	22.6 (3)
N1A—N2A—C8A—C9A	−178.14 (16)	N1B—N2B—C8B—C9B	−177.69 (16)
N2A—C8A—C9A—C14A	−179.67 (19)	N2B—C8B—C9B—C14B	176.00 (19)
N2A—C8A—C9A—C10A	1.2 (3)	N2B—C8B—C9B—C10B	−0.6 (3)
C14A—C9A—C10A—C11A	1.3 (3)	C14B—C9B—C10B—C11B	−2.8 (3)
C8A—C9A—C10A—C11A	−179.55 (17)	C8B—C9B—C10B—C11B	173.83 (17)
C15A—O3A—C11A—C10A	14.2 (3)	C15B—O3B—C11B—C10B	−2.4 (3)
C15A—O3A—C11A—C12A	−165.74 (19)	C15B—O3B—C11B—C12B	178.49 (19)
C9A—C10A—C11A—O3A	−177.20 (18)	C9B—C10B—C11B—O3B	−178.14 (17)
C9A—C10A—C11A—C12A	2.8 (3)	C9B—C10B—C11B—C12B	0.9 (3)
O3A—C11A—C12A—C13A	175.5 (2)	O3B—C11B—C12B—C13B	−179.62 (18)
C10A—C11A—C12A—C13A	−4.4 (3)	C10B—C11B—C12B—C13B	1.2 (3)
C11A—C12A—C13A—C14A	2.0 (4)	C11B—C12B—C13B—C14B	−1.5 (3)
C12A—C13A—C14A—C9A	2.1 (3)	C10B—C9B—C14B—C13B	2.5 (3)
C10A—C9A—C14A—C13A	−3.7 (3)	C8B—C9B—C14B—C13B	−174.18 (18)
C8A—C9A—C14A—C13A	177.1 (2)	C12B—C13B—C14B—C9B	−0.3 (3)

### Hydrogen-bond geometry (Å, º)

*Cg*_4_ is the centroid of the C9*B*–C14*B* ring.

**Table:**

*D*—H···*A*	*D*—H	H···*A*	*D*···*A*	*D*—H···*A*
N1*A*—H1*A*···O2*B*^i^	0.85	2.58	3.354 (2)	153
N1*B*—H1*B*···O3*A*^ii^	0.87	2.32	3.178 (3)	170
O2*A*—H2*A*···O1*A*^iii^	0.82	1.94	2.702 (2)	155
O2*A*—H2*A*···N2*A*^iii^	0.82	2.60	3.231 (2)	135
O2*B*—H2*B*···O1*B*^ii^	0.82	1.92	2.696 (2)	157
O2*B*—H2*B*···N2*B*^ii^	0.82	2.52	3.110 (2)	129
C13*B*—H13*B*···O1*A*^iv^	0.93	2.57	3.352 (3)	143
C3*B*—H3*B*···*Cg*^v^	0.93	2.70	3.604 (2)	165

Symmetry codes: (i) −*x*, −*y*+1, −*z*+1; (ii) *x*, −*y*+3/2, *z*−1/2; (iii) *x*, −*y*+1/2, *z*−1/2; (iv) *x*, *y*+1, *z*; (v) −*x*, *y*−1/2, −*z*+3/2.
